# Intervention Effects on Phonological Processing in Children With Developmental Speech and/or Language Disorder: A Systematic Review and Meta‐Analysis of Studies With Group Design

**DOI:** 10.1111/1460-6984.70252

**Published:** 2026-05-11

**Authors:** Marja Laasonen, Susana Sanduvete‐Chaves, Salvador Chacón‐Moscoso, Dina Caetano Alves, Martina Ozbič, Kakia Petinou, Anna‐Kaisa Tolonen, Krisztina Zajdó, Pauline Frizelle, Carol‐Anne Murphy, David Saldaña, Sari Kunnari

**Affiliations:** ^1^ Department of Logopedics, School of Humanities University of Eastern Finland Joensuu Finland; ^2^ Departamento de Psicología Experimental, Facultad de Psicología Universidad de Sevilla Sevilla Spain; ^3^ Departamento de Psicología Universidad Autónoma de Chile Santiago Chile; ^4^ School of Health Polytechnic Institute of Setúbal Setúbal Portugal; ^5^ Center of Linguistics, School of Arts and Humanities University of Lisbon Lisbon Portugal; ^6^ Department of Educational Studies University of Primorska, Faculty of Education, Koper‐Capodistria and LOGOS, private clinical practice Sežana Slovenia; ^7^ Department of Rehabilitation Sciences Cyprus University of Technology Limassol Cyprus; ^8^ Department of Special Education/Speech‐Language Therapy Széchenyi István University/The University of Győr Győr Hungary; ^9^ Department of Speech and Hearing Sciences University College Cork Cork Ireland; ^10^ School of Allied Health and Health Research Institute University of Limerick Limerick Ireland; ^11^ Departamento de Psicología Evolutiva y de la Educación Facultad de Psicología Universidad de Sevilla Sevilla Spain; ^12^ Research Unit of Logopedics University of Oulu Oulu Finland

**Keywords:** intervention, language disorder, meta‐analysis, phonological processing, speech disorder, systematic review

## Abstract

**Background:**

Challenges in phonological processing are prevalent in children with developmental speech and/or language disorders and are associated with their language acquisition and later reading skills. A recent systematic review and meta‐analysis from the same project of studies with a group design on the effects of interventions on output phonology, found that oral language interventions have a positive effect on output phonology, specifically speech production accuracy. These interventions are expected to impact the phonological representational level as well and, thus, also affect phonological processing skills in this population. However, no prior systematic reviews or meta‐analyses have been conducted on this topic.

**Aims:**

We conducted a systematic literature review and meta‐analysis to investigate the effectiveness of oral language interventions on children and adolescents with developmental speech and/or language disorders, focusing on phonological processing as the primary outcome measure, including phonological awareness, phonological retrieval efficiency, and short‐term memory.

**Methods and Procedures:**

The systematic review was pre‐registered (PROSPERO ID: CRD42017076075) and followed the PRISMA guidelines. A search for oral language interventions in children and adolescents with developmental speech and/or language disorders (mean age, 3–18 years) was conducted across seven databases (PubMed, Web of Science, ERIC, Cochrane Library, PsycINFO, Scopus, LLBA) for studies published between January 2006 and May 2023. We included studies with a group design and phonological processing as the outcome measure. The Cochrane risk of bias tool was used for study appraisal. Individual and average standardised mean differences *g* were used in the meta‐analysis. We also explored the influence of various moderators.

**Outcomes and Results:**

We identified 22 studies that met the inclusion criteria. Overall, the risk of bias was low to moderate. Significant and large effect sizes were found in pre‐post studies with control groups, showing improvements post‐intervention in phonological awareness, *g* = 0.710, *p* < 0.001, 95% CI [0.504, 0.917], and phonological short‐term memory, *g* = 0.785, *p* = 0.010, 95% CI [0.192, 1.279]. Some moderator variables emerged, for example, larger effects when the interventions were based on an integrated theoretical approach, applied in small groups, or when they had processing targets.

**Conclusions and Implications:**

The results demonstrate that oral language interventions can effectively improve specific phonological processing skills in children and adolescents with developmental speech and/or language disorders. The identification of key moderating factors provides valuable guidance for clinical practice.

**WHAT THIS PAPER ADDS:**

*What is already known on the subject*

- Children with developmental speech and/or language disorders often have difficulties in phonological processing that can hamper their reading development. Interventions are effective for speech production, but there are no previous systematic reviews or meta‐analyses on their effects on the phonological processing skills of children and adolescents with developmental speech and/or language disorders.

*What this paper adds to existing knowledge*

- We present empirical evidence of the effectiveness of oral language interventions in improving phonological processing (including phonological awareness and phonological short‐term memory) in children and adolescents with developmental speech and/or language disorders.

*What are the potential or actual clinical implications of this work?*

- Oral language interventions have a positive effect on the phonological processing skills of children and adolescents with developmental speech and/or language disorders. They may serve as an indirect means of support for their reading development.

## 1 Introduction

Language is the most important means of human communication, and its acquisition during the preschool period is a complex process. 7.5% of children have challenges with language development without a known cause, to the extent that it interferes with their functioning, ability to successfully participate, and engage with education (Norbury et al. 2016; Tomblin et al. 1997). Children with such developmental speech and/or language disorders can experience difficulties in various dimensions of language and its acquisition, including phonology, syntax, word finding and semantics, pragmatics and language use, as well as discourse (Bishop et al. 2017). Interventions targeting these various areas of language are manifold, but the evidence for their effect is only emerging.

The present review was conducted within the COST Action IS1406 – Enhancing children's oral language skills across Europe and beyond—a collaboration focusing on interventions for children with difficulties learning their first language (https://www.cost.eu/actions/IS1406/) (2015–2019) that aimed to evaluate the evidence base for oral language interventions for children with developmental speech and/or language disorders. The Action produced a coordinated series of systematic reviews and meta‐analyses, each focusing on a different aspect of intervention outcomes: vocabulary, morphosyntax, output phonology (speech production) (Kunnari et al. 2024), pragmatics (Jensen de Lopez et al. 2022), and intervention dosage (Frizelle et al. 2021a, 2021b). For example, Frizelle et al. (2021a) investigated the quantitative effects of dosage. They found that several dosage models yielded effective outcomes (e.g., frequent short sessions (2 min, 2/3 times a week) as well as less frequent long sessions (20 min, once a week). The effects of interventions on output phonology (e.g., speech production accuracy, speech intelligibility, and phonemic inventory) are reported in another article from this group (Kunnari 2024), which suggests positive effects on phonological accuracy. Although the COST Action formally ended in 2019, the analysis and publication of individual reviews have continued beyond the funding period. The current systematic review and meta‐analysis focuses on the effects of interventions on the phonological processing skills of children and adolescents with developmental speech and/or language disorders, which implicate the representational level to a greater extent than output phonology.

*Phonological processing* refers to the processing of the phonological or sound structure of language (Torgesen et al. 1994). It is often separated into three sub‐processes: *Phonological awareness, Phonological retrieval efficiency and Phonological (short‐term) memory. Phonological awareness* is the awareness of the sound structure of language (Swan and Goswami 1997; Witton et al. 1998). Phonological awareness tasks range from easier to more demanding based on linguistic complexity and task difficulty (Hulme et al. 2002; Stahl and Murray 1994; Yopp 1988). In general, easier tasks can require awareness of onsets and rhymes, phoneme detection and categorisation (alliteration), and naming the first or last phoneme of a word (isolation). More difficult tasks require combining phonemes into a word (blending), deleting a phoneme from a word (elision), telling the phonemes in a word (segmentation), and transposing the initial sounds of words (spoonerism) (Bus and Van IJzendoorn 1999; Gillon 2017). This hierarchy of difficulty, however, might depend on the language (Bider Petelin 2022). *Phonological retrieval efficiency* relies on fast access to phonological information in long‐term memory (Torgesen et al. 1994). Phonological retrieval efficiency is assessed with the speed and accuracy of naming matrices of symbols or objects in rapid automatised naming (RAN) or rapid alternating stimulus (RAS) tests. In the first, the class of the items to be named is constant, and in the latter, the item classes alternate (e.g., colours, letters, and digits in the same matrix) (Denckla and Rudel 1974; Wolf 1986). *Phonological (short‐term) memory* refers to the coding and temporary storage of sound‐based representations or phonological properties of stimuli (cf. the phonological*/*articulatory loop of the Baddeley and Hitch working memory framework (Baddeley 2003; Baddeley and Hitch 1974)). Phonological short‐term memory can be evaluated with memory span tasks for meaningless sequences, where the child repeats the serial order of lengthening sequences of digits or nonwords, or with nonword repetition tasks that require repetition of single items of varying syllable lengths (Gathercole 2006).

Each of these areas of phonological processing has been associated with language acquisition and developmental language difficulties. Cross‐linguistically, phonological awareness develops from larger units (syllables) to smaller ones (phonemes) in preschool and early school years, with the increasingly complex tasks mastered sequentially but correlating with each other (Anthony and Francis 2005; Gillon 2017). Performance in rhyming, detection, alliteration, blending, and elision correlates with various aspects of language, for example, receptive and expressive vocabulary, grammar, and narrative skills (Carroll et al. 2003; Hipfner‐Boucher et al. 2014; Lonigan et al. 1998). Developmental speech and/or language disorders are often associated with poor phonological awareness, spanning tasks of onset and rhymes (Briscoe et al. 2001; Fazio 1997), blending and elision (Claessen et al. 2013; Vandewalle et al. 2012), substitution (De et al. 2015), and spoonerisms (Stothard et al. 1998; Vandewalle et al. 2012). Furthermore, phonological awareness has been linked especially to emerging reading ability and accuracy (Bradley and Bryant 1978; Goswami 1993; Goswami 1999; Moll et al. 2014) and to difficulties in these areas and developmental dyslexia (Bradley and Bryant 1978; Landerl et al. 2013).

RAN also develops during preschool and early school years, and performance for different item classes correlates (Denckla and Rudel 1974; van den Bos et al. 2002; Åvall et al. 2019). Although progress in serial naming for non‐alphanumeric items (colours, objects) precedes that for alphanumeric (digits, letters) items, the former continues to develop until later middle age, and the latter is ultimately faster as opposed to the former, which progresses faster (van den Bos et al. 2002). Phonological retrieval efficiency is associated with other naming and with verbal fluency (Närhi et al. 2005) but also with, for example, semantic and syntactic receptive language (Albuquerque and Simoes 2010). Children with developmental speech and/or language disorders have been found to be slow but accurate in alphanumeric and non‐alphanumeric RAN and RAS (Claessen et al. 2013; Isoaho et al. 2016; Nicolielo and Hage 2014; Wiig et al. 2000). Phonological retrieval efficiency also predicts reading skills and fluency (Bowers et al. 1988; Moll et al. 2014; Wolf 1991) and difficulties in rapid naming tasks are associated with developmental dyslexia (Kinsbourne et al. 1991; Landerl et al. 2013).

As with the other two subcomponents of phonological processing, phonological memory capacity also develops qualitatively from preschool to early school years when children gradually move from merely storing the heard material to using strategies, such as rehearsal (Gathercole et al. 1992; Gathercole 1998). Further development in capacity continues until adolescence when the rehearsal rate increases (Gathercole et al. 2004). Performance in span and nonword repetition tasks correlates (Gathercole 2006) and phonological memory assessed with span and repetition tasks has been linked especially with the early stages of vocabulary acquisition and building of lexical knowledge (Baddeley 2003; Gathercole 2006). However, there is also evidence of a link between phonological memory and morphology and syntax (Verhagen and Leseman 2016). Difficulties in digit and nonword span and nonword repetition have been reported in many studies involving children with developmental speech and/or language disorders (Archibald and Gathercole 2006; Archibald et al. 2009; Baddeley 2003; Claessen et al. 2013; Delcenserie et al. 2021; Gathercole and Baddeley 1990; Stothard et al. 1998; Vandewalle et al. 2012). Phonological memory has also been associated with both successful reading accuracy development (Moll et al. 2014) and developmental dyslexia (Landerl et al. 2013).

Taken together, difficulties in the three areas of phonological processing are not only expected to contribute to language‐related challenges but also to be associated with the reading skills of children with developmental speech and/or language disorders. Further, it has been suggested that children with developmental speech and/or language disorders and comorbid reading difficulties experience pronounced difficulties in all areas of phonological processing compared to those with speech and language difficulties alone (De et al. 2015; Loucas et al. 2016; Vandewalle et al. 2012). A note on terminology is warranted, as the classification and labelling of developmental speech and language difficulties have evolved. In this review, we use “developmental speech and/or language disorder” as an umbrella term encompassing both developmental language disorder (DLD), as defined by CATALISE (Bishop et al. 2017), and speech sound disorder (SSD) affecting phonology (i.e., excluding motor difficulties; Stringer et al. 2024). This broad scope is motivated by two considerations: first, DLD and SSD frequently co‐occur; and second, deficits in phonological processing have been documented in both populations. Where the original studies used earlier terminology (e.g., “specific language impairment,” “language delay”), we retain these terms when reporting specific findings but subsume them under the umbrella term in our synthesis.

Interventions targeting phonology are purported to be founded on several theoretical *approaches* classified into (1) perceptually based (i.e., input‐oriented), (2) phonologically based, and (3) motorically based (i.e., output‐oriented) (Cabbage and DeVeney 2020; Rvachew and Brosseau‐Lapré 2016). The first class, input‐oriented interventions, aim to strengthen acoustic‐phonetic representations, which are especially recommended for children with phonological disorders and challenges in phonological processing (Rvachew and Brosseau‐Lapré 2016). The most commonly used input‐based interventions for phonological disorders tend to target auditory discrimination, awareness of the meaning of phonological contrasts through minimal pairs, and phonological awareness (Hegarty et al. 2018; Joffe and Pring 2008). The second class, phonologically based interventions, aim to reorganise the cognitive‐linguistic system of a child with limited phonological knowledge (Dodd et al. 2008). These interventions typically involve contrasting word pairs so that the child can recognise and produce phonemic differences that signal changes in meaning. The third class, output‐oriented interventions, aim to improve motor‐speech control (Cabbage and DeVeney 2020). For example, the traditional articulation, or motor‐based, approach emphasises articulatory placement and movement. In their systematic review, Wren et al. (2018) did not focus on the theoretical approaches underpinning interventions. Still, they found that the most frequent *targets* for intervention for speech‐sound disorder were cognitive–linguistic and output‐oriented. Thus, although it appears that input‐oriented approaches might support phonological processing in children with phonological disorders, the literature has not been summarised systematically for developmental speech and/or language disorders, nor have the three theoretical approaches been compared to each other.

The effects of interventions on output phonology are published in another systematic review and meta‐analysis (Kunnari 2024). The results indicated that interventions, in general, had a medium to large effect on phonological accuracy. Significant moderator variables and risk of publication bias were not detected. There were insufficient studies to conduct meta‐analyses of intervention effects on speech intelligibility or phonemic inventory.

To the best of our knowledge, there are no previous systematic reviews or meta‐analyses explicitly examining the effect of interventions on phonological processing in children and adolescents with developmental speech and/or language disorders. Further, of the previous reviews with a slightly differing scope, only four have included partly relevant findings. In their seminal systematic review and meta‐analysis, James Law and colleagues (1996) focused, among other domains, on speech and language therapy interventions targeting receptive phonology outcomes and found no effects of parent‐administered listening training in the one included study (Shelton et al. 1978). Cirrin and colleagues (2008) systematically reviewed language intervention practices in children with developmental speech and/or language disorders and found that targeting phonological awareness improved phonological awareness. Goldfeld et al. (2022) conducted a systematic review on oral language and early reading interventions. The authors found evidence for phonological awareness interventions improving phonological awareness in children ranging from those at risk to those experiencing oral language or reading difficulties. Recently, Tarvainen et al. (2025) conducted a meta‐analysis on interventions targeting oral language comprehension and included interventions aimed at improving general language processing skills, such as auditory processing and working memory. Although phonological processing was not specifically investigated in the meta‐analysis, the broader category of interventions aimed at language processing showed no clinically significant efficacy, and the observed effect sizes were notably smaller compared to interventions targeting language environment, specific language skills, or compensatory strategies. As language and reading development and their disorders are closely associated with developing phonological processing skills, it is vital to have a better understanding of whether, how, and when interventions have an effect on all three dimensions of phonological processing (awareness, phonological retrieval efficiency, and memory) in children with developmental speech and/or language disorders.

The current systematic review and meta‐analysis assesses and synthesises qualitative and quantitative findings of group studies on the effect of oral language interventions on the three aforementioned aspects of phonological processing in children and adolescents with developmental speech and/or language disorders. The objective of the current review is also to identify the theories and strategies underpinning the interventions, the methods of intervention used, the specific aspects targeted, the level of evidence supporting the interventions and their impact, and the role of moderators. Based on previous literature (Cirrin and Gillam 2008; Goldfeld et al. 2022), we hypothesised that of the theoretical approaches, input‐oriented interventions would be effective for phonological processing.

## 2 Materials and Methods

The Preferred Reporting Items for Systematic Reviews and Meta‐Analyses (PRISMA (Page et al. 2021) served as the framework for this systematic review, which is one of several as part of a European COST Action 1406 (Frizelle et al. 2021a; Frizelle et al. 2021b; Jensen de Lopez et al. 2022; Kunnari et al. 2024). The work was pre‐registered to a prospective international register for systematic reviews, PROSPERO (ID CRD42017076075; Kunnari et al. 2017). This review is the second of three that employ a comparable methodology, focusing on group studies of interventions for children and adolescents with developmental speech and/or language disorders, with phonological processing as the primary outcome measure. The first review focused on studies with group design and outcomes related to output phonology (Kunnari 2024). The third review focuses on single‐case study designs.

### 2.1 Study Selection

#### 2.1.1 Inclusion and Exclusion Criteria

The shared inclusion criteria for all COST Action IS1406 reviews were:

– Peer‐reviewed article published in any language since January 2006.
– Participants with a mean age of ≥3 and ≤18 years and identified as having a DLD or an equivalent term (i.e., performance falling below 1 SD in one or more domains using a standardised assessment method).
– Examined an oral language intervention, which measured outcomes in the domains of vocabulary, phonology, morpho‐syntax and/or pragmatics.

The specific inclusion criteria for the current review were:

– Peer‐reviewed Article Published in English between January 2006 and August 2022
– Any group design study that provided data on expressive phonology outcomes.
– Monolingual (i.e., at least 90% of the study participants were required to be monolingual) participants identified as having a DLD or an equivalent term (i.e., performance falling below 1 SD on one or more language domains) and/or clinically significant SSD based on articulation/phonology assessment or other standardised articulation/phonology measure.

The shared exclusion criteria for all COST Action IS1406 reviews were:

– Studies that included participants with a diagnosis of autism spectrum disorder, a hearing impairment, an intellectual disability, a brain injury, a physical disability or a learning disability.
– The specific exclusion criteria for the current review were:
– Participants with a diagnosis of CAS, childhood dysarthria or articulation disorder (i.e., motorically based disorders) according to judgment by the authors of the original articles or participants who were bi‐/multilingual.
– Studies with a single‐case design.
– Studies that provided data on phonological processing outcomes only.

#### 2.1.2 Search and Selection Procedure

Five steps were included in the study selection process:

(1) Electronic database search, title and abstract screening, screening for relevance to phonology, and full‐text screening. A thorough and systematic literature search was conducted in the following quality‐controlled databases: PubMed, Web of Science, ERIC, PsycINFO, SCOPUS, and LLBA. This search underpinned the different COST Action IS1406 reviews with various foci. Peer‐reviewed research published since January 2006 was included in the search (an initial database search covering studies published between January 2006 and December 2015, followed by three updated searches, the most recent in August 2022). For the multiple reviews focusing on various facets of oral language (i.e., vocabulary, phonology, morphosyntax, pragmatics, and dosage), the aim was to identify literature in any language related to oral language interventions for children and adolescents with primary language impairment (and related terms). Primary language impairment was characterised as a considerable delay between a child's oral language development in their first language and other children their age. Each database was searched for all combinations of the phrases describing the individuals’ age, disorder, and intervention see Supplementary material 1 for the detailed Search strategy). There were 17,005 studies found in these database searches. One author (AK) also conducted a hand search of the reference lists of all the papers included in the full‐text stage, as well as relevant systematic reviews, to find any other potentially pertinent studies. The resulting set of publications was then distributed to the relevant work packages, as shown in the flowchart. For the present review, the specific eligibility criteria permitted the inclusion of studies involving children with SSD, because phonological processing is relevant to both DLD and SSD populations. It is important to note that the present review is distinct from the output phonology review within the same COST Action programme: the current one focuses on input phonological processing (phonological awareness, phonological retrieval efficiency, and phonological short‐term memory), whereas the earlier one examines output phonology (speech production accuracy).

(2) The EPPI‐Reviewer 4 software, designed for systematic reviews, was used to upload all the citations found during the search procedure. Initially, eligibility criteria based on date, target population, level of evidence, or evaluation of intervention were screened by title and abstract. Twenty per cent of the papers found in the searches were double‐screened by two independent reviewers (C.A.M. and D.S. for the initial search and C.A.M. and P.F. for the updated search) to verify their relevance to the overall criteria. The agreement rate was 96%, and all issues were resolved by consensus throughout the process, both at this point and subsequently. A total of 1358 studies from the screening phase were included.

(3) The studies were then scrutinised for relevance to phonology, specifically focusing on the aim of the current review. The studies were reviewed to ensure they included key papers containing terms relevant to the theme (e.g., phonology), such as phoneme* OR phonolog* OR articulat* OR speech OR phonet*. One hundred per cent of the papers were screened by two impartial reviewers (S.K. and M.L.). The rate of agreement was 93%. A total of 796 papers were included for full‐text screening. The full texts of each of these papers were subsequently retrieved for a more thorough assessment and screened by the same independent reviewers who conducted the title and abstract screening (S.K. and M.L.). The rate of agreement was 94%. A total of 208 publications were subsequently included for data extraction.

(4) Full texts were further screened in the final step to include (a) publications with a specific focus on phonological processing outcomes, (b) group design, and (c) articles written in English. Studies with single‐case designs and results on output phonology were not included because our two other reviews cover these topics. Studies focusing on motorically‐based disorders (such as childhood dysarthria and childhood apraxia of speech) were also disregarded because the current review focused on intervention studies deemed appropriate for children and adolescents with phonological disorders. 5) Finally, because the authors could not find native speaker SLP‐experts for all papers written in non‐English languages, all non‐English articles were disqualified. At this point, the agreement rate was 100%, and 22 publications were included for data extraction and analysis.

### 2.2 Data Extraction

Data were extracted after in–depth reading of the full‐text articles that satisfied the inclusion criteria. Two impartial coders (S.K. and K.Z.) evaluated the quality of the included studies using the Cochrane risk of bias instrument (Higgins et al. 2017). The following factors were used to determine the risk of bias: selection bias for random sequence generation and allocation concealment, performance bias, detection bias, attrition bias, reporting bias, and other potential biases. Each item of bias was assigned a rating of low (0 points), high (2 points), or unclear (1 point), with the total risk score ranging from 0 to 14. Additionally, the fidelity of the intervention indicator was utilised to determine whether the intervention was carried out according to plan. Finally, coders engaged in a discussion to obtain consensus on any aspects for which there was disagreement.

The outcome variables were classified into phonological awareness with sub‐variables; summary when details not reported, onset‐rhyme/discrimination, alliteration/isolation, and blending/elision/segmentation, and spoonerism; classified based on (Bus and Van IJzendoorn 1999; Gillon 2017). Information necessary to calculate the effect size of each intervention was obtained (i.e., two groups: pre‐ and post‐assessment mean, standard deviation, *N* for the groups; one group: pre‐post mean and standard deviations or their differences, *N* for the group).

We included the following potential moderator variables: characteristics of the study (sample size and number of treatment groups), characteristics of the participants (socioeconomic status ‐SES‐, sex, ethnicity, age at intervention baseline, and diagnosis), and characteristics of the intervention (setting, theoretical approach, practitioner, intervention target, model of delivery, unit of allocation, level of service delivery, and use of software).

Additionally, other variables were extracted for descriptive purposes, including extrinsic characteristics (author, year of publication, and country), study characteristics (design), and intervention details (ingredients and proposed mechanism of change).

Six coders worked independently to complete the data extraction: two coders (D.C.A. and M.O.) extracted information about the extrinsic characteristics, the study, and the participants; two coders (S.K. and K.Z.) extracted information about the intervention, and two coders (M.L. and K.P.) extracted information about the outcome measures. A consensus served to settle disagreements. See Supplementary material 2 for the Excel database used in the meta‐analyses.

### 2.3 Data Analysis

SPSS (version 29) was used to calculate the intraclass correlation coefficient (ICC) to study the level of inter‐coder reliability. Values of 0.7 or higher were considered adequate (Koo and Li 2016). Excel was used to support data extraction, and Comprehensive Meta‐Analysis (version 4) was used to conduct the data analysis. Hedges’ *g* were obtained as individual effect sizes for each sample and each dependent variable. When studies included more than one indicator for the same sample, a composite value was obtained following the procedure outlined by Borenstein and colleagues (Borenstein et al. 2009).

Given that some studies presented samples of fewer than 20 participants, Hedges’ *g* was obtained as an average effect size. A value of around ±0.2 was considered a small effect size; around ±0.5, medium; and around ±0.8, large (Cohen 1988). *p* values >0.05 were considered significant.

Homogeneity was studied based on the statistics *Q* (*p* values below 0.05 would imply heterogeneity), *I^2^* (around 25% being low heterogeneity, around 50% medium, and around 75% high), and the prediction interval (wide being heterogeneous; i.e., ranging from small to high effect sizes).

When there were indicators of heterogeneity, the possible influence of all the potential moderator variables was studied, using ANOVA F when the moderator variable was categorical, and using meta‐regression when the moderator variable was quantitative.

Not all moderator variables yielded results in all analyses due to a lack of information and/or data variability. Moderator variables were studied when there were at least four individual effect sizes available based on the dependent variable studied and the design.

To study the risk of publication bias, we obtained: (a) funnel plots (when the estimated diamond represented with black colour was close to the observed diamond represented with white colour, there was low risk of publication bias) (Borenstein 2019; Borenstein et al. 2009); and (b) Egger's test of the intercept (B_0_), where a non‐significant result (*p* < 0.05) is interpreted as absence of important publication bias.

## 3 Results

### 3.1 Study Selection

**FIGURE 1:**
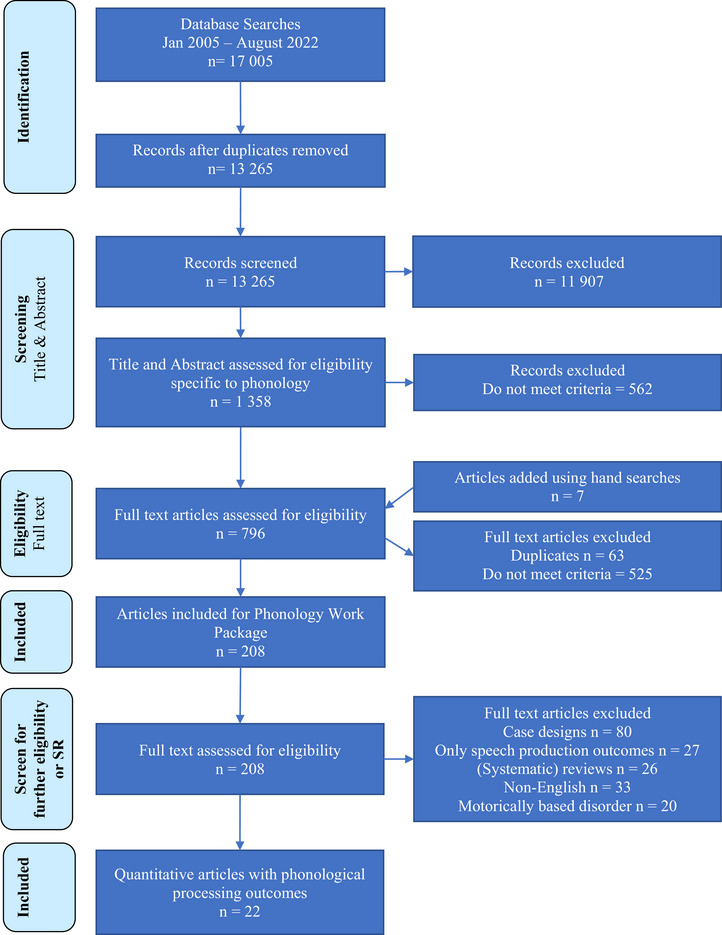
Preferred reporting items for systematic reviews and meta‐analyses flowchart of the process of study selection. SR, systematic review.

Figure 1 presents the flow diagram illustrating the selection process of studies at each level, culminating in the 22 publications ultimately included in this study.

### 3.2 Study Characteristics

Tables 1 and 2 present detailed information for each of the primary studies included. The most frequent country of publication (see Table 1) was the United Kingdom (27.3%), followed by Canada and the USA (18.2%). The total number of participants was 1279. When the socioeconomic status of the families was reported (in 36.4% of the studies), it was mainly defined as mixed (87.5% of the cases reported). According to the studies that reported SES information (72.7%), all except one presented a higher number of males than females, with the percentage of males ranging from 44.4% to 81.6%. The percentage of Caucasians was reported only in 18.2% of the occasions, ranging from 48% to 82.5%. The age of the participants ranged from 48 to 130 months (*M* = 67.09). In most cases (68.2%), the diagnosis included both receptive and expressive impairment.

**TABLE 1:** Characteristics of the primary studies included (extrinsic and substantive, referring to the participants and the intervention).

Author, year	Country	N	SES	% male	% Cauc	Age (months)	Diagnosis	Design	Outcome	Setting	Theoretical approach
Bishop et al. 2006	UK	33				130	R	RCT	PA (OR/D)	PS/SS	Auditory perceptual
Buschmann et al. 2015	Germany	43	MX	52.2		51	E	RCT	PSTM	Hospital	Environmental
Carson 2020	Australia	24	MX	54.2		57	MX	RCT	PA (OR/D, A/I)	P/N/K	Integrated
Chen and Lin 2022	Taiwan	49				69	MX	QE	PA (OR/D)	P/N/K/PS	Integrated
Fricke et al. 2013	UK	180				48	Other	RCT	PA (A/I)	P/N/K	
Grawburg and Rvachew 2007	Canada	30	MX			57.3	E	Cohort	PA (OR/D)	P/N/K	Integrated
Gross et al. 2010	Germany	18		66.7		52	MX	Cohort	PSTM	Hospital	Auditory perceptual
Henry et al. 2022	UK	32				96.2	MX	RCT	PSTM	PS/SS	Cognitive‐linguistic
Hesketh et al. 2007	UK	42		81		51	E	RCT	PA (A/I, B/D/S/S)	Home	Integrated
Holmes et al. 2015	UK	27		55.6		117	MX	QE	PSTM	PS/SS	WMT
Hund‐Reid and Schneider 2013	Canada	37	MX	73	49	65.5	MX	RCT	PA (A/I, B/D/S/S)	P/N/K	Integrated
Loeb et al. 2009	USA	103	MX	66.7	48	89	MX	QE	PA (B/D/S/S)	PS/SS	Auditory perceptual
McCartney et al. 2011	UK	69		81.6		105	MX	RCT	PA (OR/D, A/I, B/D/S/S), PRE	PS/SS	
McNamara John et al. 2008	Canada	26	MD	69.2		49	MX	RCT	PA (OR/D, A/I)	Public clinic	Integrated
Mota et al. 2009	Brazil	18		44.4		60	E	Cohort	PA (summary)	Public clinic	Integrated
Munro et al. 2008	Australia	17		76.5		67	MX	Cohort	PA (OR/D, A/I)		Integrated
Park et al. 2014	USA	50		60	82	93.5	MX	Cohort	PSTM	UC	Integrated
Ritter et al. 2013	USA	64	MX	77.3	82.5	91.5	MX	QE	PA (B/D/S/S)	P/N/K/PS/SS	Integrated
Roden et al. 2019	Germany	101		62.6		54	MX	RCT	PSTM, PA (OR/D)	P/N/K	Integrated
Rvachew and Brosseau‐Lapré 2015	Canada	85		70.7		53	E	RCT	PA (OR/D)		Integrated
Van et al. 2009	USA	52				62	MX	Cohort	PSTM	P/N/K	Cognitive‐linguistic
Wake et al. 2013	Australia	179	MX	68		49.5	MX	RCT	PA (summary)	Home	

Abbreviations: A/I, Alliteration/Isolation; B/D/S/S, Blending/Deletion/Segmentation/Spoonerism; Cauc, Caucasian; E, expressive; MD, Middle; MX, Mixed; N, sample size; OR/D, Onset‐Rhyme/Discrimination; P/N/K, Preschool/Nursery/Kindergarten; PA, Phonological Awareness; PRE, Phonological Retrieval Efficiency; PS, Primary School; PSTM, Phonological Short‐Term Memory; QE, Quasi‐Experiment; R, receptive; RCT, Randomised Controlled Trial; SES, SocioEconomic Status; SS, Secondary School; UC, University Clinic; UK, United Kingdom; USA, United States of America; WMT, Working Memory Training.

**TABLE 2:** Other characteristics related to the intervention and the main results found.

Author, year	Practitioner	Intervention target	Delivery model	Unit of allocation	Level of service	Software	Ingredients	Mechanism of change
Bishop et al. 2006	Non‐specialist teachers	No speech/Processing	Indirect	One‐to‐one	Universal	Yes	Implicit	Specified
Buschmann et al. 2015	Parent/carer	Speech and language	Indirect	One‐to‐one	Universal	No	Specified	Specified
Carson 2020	Non‐specialist teachers/students	No speech/Processing	Direct	Small group	Targeted	Yes	Implicit	Specified
Chen and Lin 2022	Students	Processing	Direct	Small group and one‐to‐one	Targeted	Yes	Implicit	Inferable
Fricke et al. 2013	Teaching assistants	Speech and language	Direct	Small group and one‐to‐one	Universal	No	Implicit	Specified
Grawburg and Rvachew 2007	Students	Speech and language	Direct	One‐to‐one	Specialist	Yes	Implicit	Non‐ specified
Gross et al. 2010	Others	Speech and language/processing	Direct	One‐to‐one	Universal	No	Unclear	Non‐ specified
Henry et al. 2022		Processing	Direct	One‐to‐one	Specialist	No	Implicit	Inferable
Hesketh et al. 2007		Speech and language	Direct	One‐to‐one	Specialist	No	Specified	Inferable
Holmes et al. 2015	Others	Speech and language/processing	Direct	Small group	Targeted	Yes	Implicit	Inferable
Hund‐Reid and Schneider 2013	Teaching assistants	Speech and language/processing	Direct	Small group	Targeted	No	Implicit	Specified
Loeb et al. 2009	Speech‐language pathologist/therapist	Speech and language	Direct	Small group and one‐to‐one	Specialist	Yes	Implicit	Specified
McCartney et al. 2011	Non‐specialist teachers/teaching assistants	Speech and language/processing	Indirect	Large group	Targeted	No	Unclear	Non‐ specified
McNamara John et al. 2008	Speech‐language pathologist/therapist	Speech and language	Direct	One‐to‐one	Specialist	No	Implicit	Inferable
Mota et al. 2009		Speech and language	Direct	One‐to‐one	Targeted	No	Unclear	Inferable
Munro et al. 2008	Speech‐language pathologist/therapist	Speech and language	Direct	One‐to‐one	Specialist	No	Implicit	Non‐ specified
Park et al. 2014	Students	No speech/Processing	Direct	One‐to‐one	Specialist	No	Specified	Specified
Ritter et al. 2013	Speech‐language pathologist/therapist	Speech and language	Direct	Small group	Specialist	No	Specified	Non‐ specified
Roden et al. 2019	Non‐specialist teachers	No speech/Processing	Direct	Small group	Universal	No	Implicit	Specified
Rvachew and Brosseau‐Lapré 2015	Speech‐language pathologist/therapist	Speech and language	Direct	One‐to‐one	Specialist	No	Implicit	Inferable
Van et al. 2009	Others	Speech and language/processing	Direct	Small group	Targeted	No	Unclear	Inferable
Wake et al. 2013	Others	Speech and language	Direct/indirect	One‐to‐one	Targeted	No	Unclear	Non‐ specified

Most of the studies were randomised controlled trials (54.5%), followed by cohort studies (27.3%). The context in which the intervention was given was mainly educational (65% of the studies reported this information). In most cases (63.2% of the studies with reported information), the intervention was based on an integrated theoretical approach.

In most studies (52.6% of the studies that reported this information), the practitioner who delivered the intervention was a non‐specialist (teachers in 21% of cases, teacher assistants in 10.5%, students in 15.8% and parents or carers in 5.3%); 26.3% of the interventions were implemented by specialists (see Table 2). The most frequent intervention target was speech and language (72.7%), followed by processing (50%). The prototype intervention was characterised by a direct model of delivery (86.4%), applied individually (68.2%) or in small groups (40.9%), with a specialist (40.9%) or targeted (36.4%) level of service, and without the use of software (72.7%). The ingredients were specified in only 18.2% of the studies; in most cases, they were implicit in the articles (59.1%), and the mechanism of change was mentioned in 36.4% of the studies (although it was inferable in 36.4% of these).

### 3.3 Risk of Bias

Inter‐coder reliability was adequate with an ICC of 0.913, 95% CI [0.885, 0.935]. Table 3 presents the risk of bias analysis. The risk of bias was scored on a scale ranging from minimal risk (1) to high risk (11), with a mean (M) of 5.05 and a standard deviation (SD) of 2.94. Generally, the studies showed a low to moderate risk of bias. Specifically, in most studies, items 1 (random sequence generation), 4 (detection bias), 5 (attrition bias), and 6 (reporting bias) were scored as low risk (median of 0). In contrast, items 2 (allocation concealment), 3 (performance bias) and 7 (other potential bias) were more often at a medium risk (median of 1). Furthermore, while many studies (45.4%) reported fidelity measures, a relatively large percentage (40.9%) did not provide this information.

**TABLE 3:** Risk of bias study of the primary studies included (Higgins et al. 2017) and fidelity measures.

Author, year	Random	Allocation	Perform	Detection	Attrition	Reporting	Other	Fidelity
Bishop et al. 2006	Low risk	Low risk	Unclear	Unclear	Low risk	Low risk	Unclear	Yes, implicit
Buschmann et al. 2015	Low risk	Low risk	Low risk	Low risk	Low risk	Low risk	High risk	Yes, implicit
Carson 2020	Low risk	Unclear	High risk	Unclear	Low risk	Low risk	Unclear	No
Chen and Lin 2022	Low risk	Unclear	High risk	Low risk	Low risk	Low risk	Unclear	Yes, explicit
Fricke et al. 2013	Low risk	Unclear	Unclear	Unclear	Low risk	Low risk	Unclear	Yes, explicit
Grawburg and Rvachew 2007	High risk	High risk	High risk	High risk	Unclear	Low risk	High risk	No
Gross et al. 2010	Unclear	Unclear	High risk	High risk	Low risk	Low risk	Unclear	No
Henry et al. 2022	Low risk	Low risk	High risk	Low risk	Low risk	Low risk	Low risk	No
Hesketh et al. 2007	Low risk	Low risk	Low risk	Low risk	Low risk	Low risk	Unclear	No
Holmes et al. 2015	High risk	High risk	Unclear	Low risk	High risk	Low risk	Unclear	No
Hund‐Reid and Schneider 2013	Low risk	Low risk	Unclear	Low risk	Low risk	Low risk	Unclear	Yes, explicit
Loeb et al. 2009	High risk	High risk	High risk	Low risk	Low risk	Low risk	Unclear	Yes, explicit
McCartney et al. 2011	Unclear	Unclear	Unclear	Low risk	Low risk	Low risk	Unclear	No
McNamara John et al. 2008	Low risk	Unclear	Low risk	Unclear	Low risk	Low risk	High risk	Yes, implicit
Mota et al. 2009	Unclear	Unclear	Unclear	Unclear	High risk	High risk	High risk	No
Munro et al. 2008	High risk	High risk	Low risk	Low risk	Low risk	Low risk	High risk	Yes, explicit
Park et al. 2014	Low risk	Unclear	Unclear	Low risk	Low risk	Low risk	High risk	Yes, explicit
Ritter et al. 2013	Low risk	Unclear	Unclear	Low risk	Unclear	Low risk	High risk	Yes, explicit
Roden et al. 2019	Low risk	Unclear	Unclear	Unclear	Unclear	Unclear	Unclear	No
Rvachew and Brosseau‐Lapré 2015	Low risk	Low risk	Low risk	Low risk	Low risk	Low risk	High risk	Yes, explicit
Van et al. 2009	High risk	High risk	High risk	High risk	Unclear	Low risk	High risk	No
Wake et al. 2013	Low risk	Low risk	Unclear	Low risk	Low risk	Low risk	High risk	Yes, explicit

*Note*: Random = Selection bias: Random sequence generation; Allocation = Selection bias: Allocation concealment; Perform = Performance bias: Blinding of participants and personnel; Detection = Detection bias: Blinding of outcome assessment; Attrition = Attrition bias: Incomplete outcome data; Reporting = Reporting bias: Selective reporting; Other = Other sources of bias.

### 3.4 Summary of Quantitative Findings

Table 4 summarises the results obtained in terms of average effect size, heterogeneity, significant moderator variables and publication bias based on the different outcomes studied in the various designs. See Supplementary material 3 for the Statistical data used to obtain the individual and average effect sizes and Supplementary material 4 for the Detailed information about the results obtained.

**TABLE 4:** Results in the quantitative integration.

Outcome	Design	Number of studies	*g* (*p*) [95% CI]	Prediction interval	*Q* (*p*)	*I^2^*	Significant moderator variables	B_0_ (*p*)
**Phonological awareness (GLOBAL)**	Unmatched pre‐post	18	0.710 (< 0.001) [0.504, 0.917]	0.100, 1.321	28.299 (0.042)	39.778	Sample size, *z* = −2.95, *p* = 0.003; percentage Caucasian, *z* = 3.33, *p* < 0.001; theoretical approach, *Q*(1) = 6.523, *p* = 0.011; intervention target, *Q*(3) = 13.792, *p* = 0.003; unit of allocation, *Q*(2) = 12.913, *p* = 0.002; software, *Q*(1) = 4.134, *p* = 0.042	1.254 (0.137)
**Phonological awareness (onset rhyme/discrimination)**	Unmatched pre‐post	11	0.573 (< 0.001) [0.321, 0.825]	No dispersion of true effects	7.114 (0.715)	0	Homogeneity	0.972 (0.450)
**Phonological awareness (alliteration/isolation)**	Unmatched pre‐post	5	0.961 (0.002) [0.341, 1.580]	−1.239, 3.161	20.374 (< 0.001)	80.368	Diagnosis, *Q*(2) = 7.723, *p* = 0.021; intervention target, *Q*(2) = 6.569, *p* = 0.037; unit of allocation, *Q*(2) = 7.843, *p* = 0.020; level of service delivery, *Q*(2) = 7.843, *p* = 0.020	2.964 (0.250)
**Phonological Awareness (Blending/Elision/ Segmentation/Spoonerism)**	Unmatched pre‐post	7	1.019 (< 0.001) [0.527, 1.510]	−0.620, 2.657	28.704 (< 0.001)	79.097	Sample size, *z* = −5.05, *p* < 0.001; age, *z* = −2.71, *p* < 0.007; setting, *Q*(3) = 17.080, *p* = 0.001; practitioner, *Q*(2) = 23.475, *p* < 0.001; theoretical approach, *Q*(1) = 6.153, *p* = 0.013; intervention target, *Q*(2) = 19.841, *p* < 0.001; unit of allocation, *Q*(2) = 10.974, *p* = 0.004; level of service delivery, *Q*(1) = 9.967, *p* = 0.002	11.277 (0.044)
**Phonological awareness (summary)**	Unmatched post	2	0.044 (0.935) [−1.014, 1.102]	Insufficient number of studies	4.387 (0.036)	77.205	Insufficient number of studies	
**Phonological awareness (GLOBAL)**	Matched pre‐post	3	0.719 (0.112) [−0.165, 1.586]	−10.146, 11.567	22.537 (< 0.001)	91.126	Insufficient number of studies	5.965 (0.315)
**Phonological awareness (alliteration/isolation)**	Matched pre‐post	2	0.674 (0.169) [−0.286, 1.634]	Insufficient number of studies	13.149 (< 0.001)	92.395	Insufficient number of studies	
**Phonological short‐term memory**	Unmatched pre‐post	5	0.785 (0.010) [0.192, 1.279]	−1.370, 2.941	21.044 (< 0.001)	80.992	Non‐significant moderator variables	1.123 (0.898)
	Unmatched post	1	0.830 (0.008) [0.213, 1.447]	Insufficient number of studies				
	Matched pre‐post	2	1.974 (0.133) [−0.604, 4.552]	Insufficient number of studies	26.068 (< 0.001)	96.164	Insufficient number of studies	
**Phonological retrieval efficiency**	Matched pre‐post	1	−0.025 (0.843) [−0.273, 0.223]	Insufficient number of studies				

*Note*: B_0_ = Egger's test for publication bias analysis.

**FIGURE 2:**
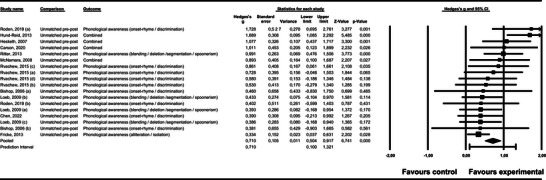
Forest plot for phonological awareness in the studies with unmatched pre‐post designs.

*Phonological Awareness (including summary, onset‐rhyme/discrimination, alliteration/isolation, and blending/deletion/segmentation/spoonerism)*. Figure 2 presents the forest plot for phonological awareness in the studies with unmatched pre‐post designs. All the studies demonstrated a greater improvement in the treatment group compared to the control group. The average effect size was medium to large and statistically significant, *g* = 0.710, *p* < 0.001. 95% CI [0.504, 0.917]. The prediction interval presented positive effects [0.100, 1.321], with specific heterogeneity from small to large effects. Other indicators of this heterogeneity include the significant Q(17) = 28.299, *p* = 0.042, and the low to medium *I* ^2^ = 39.778.

Specifying the type of phonological awareness reported, in onset rhyme/discrimination, the average effect size based on the 11 individual effect sizes available was medium and significant, *g* = 0.573, *p* < 0.001, 95% CI [0.321, 0.825] and without dispersion (all the studies shared a statistically common effect size, so prediction interval was not reported); in alliteration/isolation, a large and significant effect was found, *g* = 0.961, *p* = 0.002, 95% CI [0.341, 1.580], with high heterogeneity (the prediction interval was −1.239, 3.161, *Q*(4) *=* 20.374*, p* < 0.001, and *I* ^2^ = 80.368). Finally, in blending/elision/segmentation/spoonerism, the average effect size was also large and significant, *g* = 1.019, *p* < 0.001, 95% CI [0.527, 1.510], with high heterogeneity (the prediction interval was −0.620, 2.657, *Q*(6) *=* 28.704*, p* < 0.001, and *I* ^2^ = 79.097).

The two studies with an *unmatched post‐design* reported phonological awareness (summary). They yielded a small and non‐significant effect size, *g* = 0.044, *p* = 0.935, 95% CI [1.014, 1.102] with high heterogeneity, *Q*(1) *=* 4.387*, p* = 0.036; and *I* ^2^ = 77.205.

The three studies with *matched pre‐post design*, when all types of phonological awareness were considered, yielded a large non‐significant effect, *g* = 0.719, *p* = 0.112, 95% CI [−0.165, 1.586]. The heterogeneity was high, with a prediction interval of −10.146, 11.567, *Q*(2) *=* 22.537*, p* < 0.001, and *I* ^2^ = 91.126 (high). Taking into account exclusively the two studies that reported alliteration/isolation, the average effect was medium‐large and non‐significant, *g* = 0.674, *p* = 0.169, 95% CI [−0.286, 1.634], and the heterogeneity was high, *Q*(1) *=* 13.149*, p* < 0.001, and *I* ^2^ = 92.395.

The moderator variables that significantly influenced the effect size in the *unmatched pre‐post design*, considering phonological awareness as a whole, were the sample size, *z* = −2.95, *p* = 0.003 (when the sample size was smaller, the effect size was greater); the theoretical approach, *g* = 0.917 (large effect) for the 12 studies based on an integrated theoretical approach, *g* = 0.406 (medium) for the five studies with an auditory theoretical approach; the intervention target, *g* = 1.689 (large effect) for the study that had speech and language targets and processing targets, *g* = 0.857 (large) for the five studies without speech or processing targets, *g* = 0.562 (medium) for the eleven studies with speech and language targets, and *g* = 0.390 (medium) for the study with processing targets; the unit of allocation, *g* = 1.192 (large effect) for the five studies with small groups, *g* = 0.762 (large) for the eight studies where the intervention was individual, and *g* = 0.370 (small‐medium) for the five studies that alternated between small groups and one‐to‐one interventions; and software, *g* = 0.870 (large effect) in the eleven studies that did not use software, *g* = 0.454 (medium) in the seven studies with software.

For alliteration/isolation, the influencing moderator variables were the diagnosis, *g* = 1.262 (large effect) for the three studies whose participants presented a mixed (receptive and expressive) diagnosis, *g* = 0.989 (large) in the case of the study with an expressive diagnosis, and *g* = 0.334 (medium) for the study with another diagnosis; intervention target, *g* = 1.730 (large effect) for the study that had speech and language targets and processing targets, *g* = 0.808 (large) for the three studies with speech and language targets, and *g* = 0.421 (medium) for the study without speech/processing targets; the unit of allocation, with the same influence as when global phonological awareness was studied, *g* = 1.142 (large effect) for the two studies with small groups, *g* = 1.125 (large) for the two studies with one‐to‐one intervention, and *g* = 0.334 (small) for the study that applied both small groups and one‐to‐one interventions; and the level of service, showing large effect for the two studies with targeted (*g* = 1.142) and the two studies with specialist (*g* = 1.125) levels, and small‐medium for the study with universal level (*g* = 0.334).

Finally, when outcomes related to blending/elision/segmentation/spoonerisms only were considered, the resultant influencing moderator variables were the sample size (a lower number of participants resulted in a larger effect); age (younger children resulted in a larger effect); setting, *g* = 1.978 (large effect) in the two studies with preschool/nursery/kindergarten, *g* = 1.165 (large) in the study where the intervention took place at home, *g* = 0.991 (large) in the school context from preschool to secondary; and *g* = 0.405 (medium) for primary/secondary school; practitioner, *g* = 2.425 (large effect) for the study with a non‐specialist teacher, *g* = 1.647 (large) for the study with a teaching assistant, and *g* = 0.564 for the four studies with a speech‐language pathologist/therapist; theoretical approach, *g* = 1.468 (large) for the three studies with integrated theory, *g* = 0.405 (medium) for the three studies based on auditory perceptual theory; intervention target, *g* = 2.425 (large) for the study without speech or processing targets, *g* = 1.647 (large) for the study with speech and language, and processing targets, and *g* = 0.661 (medium‐large) for the five studies with exclusively speech and language targets; unit of allocation, *g* = 1.630 (large) for the intervention implemented one‐to‐one, *g* = 1.630 (large) for the three studies with small groups, and *g* = 0.405 (medium) for the three studies that alternated between one‐to‐one interventions and small groups; and level of service delivery, *g* = 1.978 (large) for the two studies with targeted level, and *g* = 0.661 (medium‐large) for the five studies with specialist level.

**FIGURE 3:**
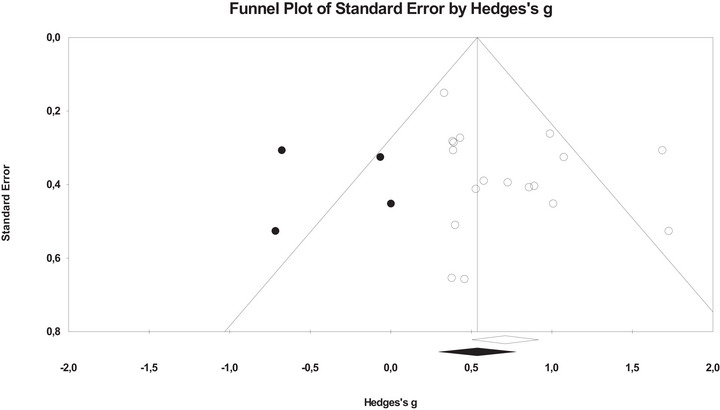
Funnel plot for phonological awareness in the studies with unmatched pre‐post designs.

Referring to publication bias, Figure 3 presents the funnel plot obtained from studies that reported phonological awareness (all types) using an *unmatched pre‐post design*. Although the estimated effect size differed slightly from the observed effect size, it remained medium. Additionally, Egger's test yielded a non‐significant result, *b_0_* = 1.254, *p* = 0.137, so we could conclude that publication bias is not an essential problem in the case of these studies.

In the other two designs (*unmatched post* and *matched pre‐post*), and when specific types of phonological awareness were considered, the results also indicated an absence of publication bias risk, with a non‐significant Egger's test and similar observed and estimated effect sizes in the funnel plot. Only when we studied blending/elision/segmentation/spoonerism (using an *unmatched pre‐post design*) was Egger's test significant *(b_0_* = 12.377, *p* = 0.044). Nevertheless, the funnel plot showed very similar observed and estimated effect sizes.

**FIGURE 4:**
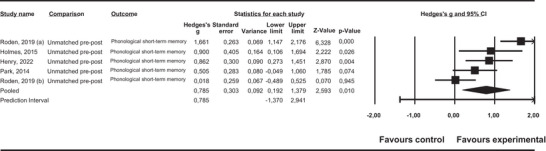
Forest plot for phonological short‐term memory in the studies with unmatched pre‐post designs.

*Phonological short‐term memory*. Regardless of the type of design, the average effect sizes were large and statistically significant, and the heterogeneity of the individual effect sizes was large (see Table 4). Figure 4 represents the forest plot for phonological short‐term memory in the studies with unmatched pre‐post designs.

None of the moderator variables studied was statistically significant, partly due to the small number of studies identified. There was no important risk of publication bias.

*Phonological retrieval efficiency*. Only one study reported results for this variable, so it could not be included in a meta‐analysis. The individual effect size was small and non‐significant (see Table 4).

## 4 Discussion

We conducted a systematic literature review and meta‐analysis of studies with a group design on oral language interventions for children and adolescents with developmental speech and/or language disorders, focusing on phonological processing (awareness, phonological retrieval efficiency, or short‐term memory) as the outcome measure. Based on a recent systematic review and meta‐analysis of studies with a group design (Kunnari et al. 2024), it was found that interventions have a positive effect on output phonology, specifically improving speech production accuracy. Interventions are also expected to affect the representational level and, thus, phonological processing skills; however, there are no previous systematic reviews or meta‐analyses on the subject. Here, a total of 22 studies were identified as meeting study inclusion criteria, and all of them were meta‐analysed. Overall, the findings of this review indicated that interventions were effective and there was no risk of publication bias. A more detailed description is presented below.

In brief, the key messages for practitioners from this review are as follows. First, oral language interventions can improve phonological awareness in children with developmental speech and/or language disorders, with moderate‐to‐large effect sizes. Second, there is evidence that phonological short‐term memory may also improve following intervention, although the evidence base is more limited. Third, small‐group delivery appears particularly effective for phonological awareness outcomes. Fourth, the current evidence on phonological retrieval efficiency is too limited to draw conclusions; the absence of evidence should not be interpreted as evidence that this skill cannot be improved through intervention.

### 4.1 Phonological Awareness

Medium to large and significant effect sizes were observed, with improvements being greater in the intervention groups. Specific heterogeneity was noted, and the absence of problems with publication bias was confirmed. Several influencing moderator variables were identified, including the theoretical approach, the intervention target, and the unit of allocation.

#### 4.1.1 Phonological Awareness (Summary, i.e., Details Not Reported)

Non‐significant small intervention effects emerged when comparing the post‐performance of intervention and control groups, with results being heterogeneous (Mota et al. 2009; Wake et al. 2013). This suggests a non‐significant effect in favour of the intervention groups on the phonological awareness summary variable. Nevertheless, given the low number of studies found, this conclusion should not be considered definitive. In fact, one of the studies (Wake et al. 2013) yielded an individual medium and significant effect size. The average effect size was small and non‐significant because, in Mota et al. (2009), the result was opposite to the expected one (the results were better in the control group). This could be due to a superior performance or baseline measures for the control group before the intervention (which we cannot determine because pre‐test measures were not reported).

#### 4.1.2 Phonological Awareness (Onset‐Rhyme/Discrimination)

Pre‐ and post‐performance of intervention and control groups differed significantly with a medium effect size, the results being homogenous with no risk of publication bias (Bishop et al. 2006; Carson 2020; Yi et al. 2022; McNamara John et al. 2008; Roden et al. 2019; Rvachew and Brosseau‐Lapré 2015). This suggests a significant and general effect of the intervention on this least demanding aspect of phonological awareness (onset‐rhyme/discrimination).

#### 4.1.3 Phonological Awareness (Alliteration/Isolation)

Significant and large effects emerged when comparing the pre‐ and post‐performance of intervention and control groups (Carson 2020; Fricke et al. 2013; Hesketh et al. 2007; Hund‐Reid and Schneider 2013; McNamara John et al. 2008). Observed heterogeneity was explained by diagnosis (all significant but most effective for mixed ‐receptive and expressive‐ diagnosis, followed by “expressive”, and least effective for “other”), intervention target (most effective with “speech and language and processing targets”, followed by “speech and language targets”, and least effective for “no speech processing targets”), unit of allocation (more effective for small groups, followed by one‐to‐one; and least effective when beginning with a small group and finishing in one‐by‐one sessions), and level of service delivery (less effective for “universal”). Sample size, SES, age, setting, practitioner, and software did not have a significant moderating role. The role of the number of treatment groups (not variability), gender (insufficient data), ethnicity (insufficient data), theoretical approach (not variability), and model of delivery (not variability) could not be investigated. This suggests a significant effect of the intervention on phonological awareness (alliteration/isolation), especially for children and adolescents with mixed or expressive diagnoses, targeting speech and language and processing, in small groups, rather than through universal delivery of service. We hypothesise that this is due to the intervention being individualised and to its support for learning from others. When comparing the pre‐ to post‐performance of the intervention group, the effect was medium‐large but not significant, with results heterogeneous (McCartney et al. 2011; Munro et al. 2008).

#### 4.1.4 Phonological Awareness (Blending/Elision/Segmentation/Spoonerism)

Significant and large effect emerged when comparing the pre‐ and post‐performance of intervention and control groups (Carson 2020; Hesketh et al. 2007; Hund‐Reid and Schneider 2013; Loeb et al. 2009; Ritter et al. 2013). Observed heterogeneity was explained by sample size (lower number of participants, larger effect), age (less effective for older), setting (all significant but more effective in preschool, nursery and kindergarten), practitioner (all significant but more effective when applied by non‐specialists), theoretical approach (both theories significant but more effective when based on an integrated view), the intervention target (all significant but more effective when processing targets plus non‐speech or speech and language targets), unit of allocation (the most effective in small groups, followed by individually, last joining small groups and individual intervention), and level of service delivery (targeted more effective than specialist). Sex, ethnicity, diagnosis, and software did not have a moderating role. The role of participating groups (all more than one), SES (all mixed) and model of delivery (all direct) could not be investigated. After correcting for the observed risk of publication bias, the intervention still had a large effect. This suggests a significant effect of the intervention on phonological awareness (blending/elision/segmentation/spoonerism), especially for preschool, nursery and kindergarten children in interventions applied by non‐specialists, in small groups, with an integrated theoretical approach, targeted in processing plus non‐speech or speech and language, and with targeted delivery of service. There was only one study comparing the pre‐ and post‐performance of the intervention group alone, which obtained a non‐significant and small effect size (McCartney et al. 2011).

### 4.2 Phonological Retrieval Efficiency

A meta‐analysis was not possible since only one study reported phonological retrieval efficiency as the outcome. However, in this study, the effect of the intervention was non‐significant for within‐group pre‐post comparison (McCartney et al. 2011). This suggests a non‐significant effect of the intervention on phonological retrieval efficiency.

### 4.3 Phonological Memory

Three studies investigated the effect of the intervention on phonological memory in between‐groups pre‐post comparison (Holmes et al. 2015; Park et al. 2014; Roden et al. 2019). The effect size was medium to large and significant, and the observed heterogeneity was not explained by any of the moderator variables that could be studied. Only one study did a post‐comparison between intervention and control groups (Buschmann et al. 2015) where the effect of the intervention was again statistically significant, with a large effect size. For within‐group pre‐post comparison, the effect of the intervention was large and statistically significant (Gross et al. 2010; Van et al. 2009). Again, the heterogeneity of the results was high, but the small number of variables prevented moderator analysis.

Taken together, oral language intervention has an effect on phonological processing in the areas of phonological awareness and phonological memory. We were unable to verify the effect on phonological retrieval efficiency. This last analysis included one study comparing the pre‐ and post‐performance of a single group; thus, the results cannot be considered definitive. However, this systematic review and meta‐analysis showed that oral language interventions have an effect on phonological processing skills at the representational level in children and adolescents with developmental speech and/or language disorders. As difficulties in phonological processing are not only expected to contribute to language‐related challenges but are also associated with the reading skills of children and adolescents with developmental speech and/or language disorders, the results of the current study suggest that supporting language might also help reading acquisition.

As language and reading development, along with their challenges, are closely associated with developing phonological processing skills, it is vital to better understand how and when interventions affect the dimensions of phonological processing in children and adolescents with developmental speech and/or language disorders. Our moderator analyses can shed light on this knowledge area. None emerged as significant for phonological memory, and for phonological retrieval efficiency, the moderator effects could not be investigated. However, for the less demanding/complex skills of phonological awareness (alliteration/isolation), those with mixed or expressive diagnoses benefited the most, receiving intervention that targeted speech and language and processing, in small groups, rather than intervention received at a universal level. For the more demanding areas of phonological awareness (blending, elision/segmentation/spoonerism), intervention was beneficial, especially for younger children in preschool, nursery and kindergarten settings, when applied by non‐specialists, in small groups, involving an integrated theoretical approach, targeting processing plus non‐speech or speech and language, and with targeted delivery of service.

Taken together, these results suggest that the delivery format is a crucial factor influencing intervention outcomes, with small groups being superior. The level of service delivery should be above universal, and the intervention should target input phonological processing and speech and language skills. For the more demanding phonological awareness processing, integrated approaches yield the best effects. Our findings on age and setting further reinforce the importance of early intervention. The unexpected finding regarding the practitioner may reflect variations in intervention intensity, the naturalness of the environment, or the integration of phonological activities in educational contexts. Alternatively, the finding may suggest that non‐specialists can deliver structured programmes effectively. For phonological short‐term memory, no significant moderators were identified, which suggests that these outcomes are less susceptible to contextual and methodological variation. Alternatively, the limited number of studies prevented the detection of moderating effects. However, this pattern of moderator influence across phonological processing domains suggests a need to consider specific outcome targets when designing and implementing interventions.

An important consideration when interpreting these findings concerns the heterogeneity of intervention approaches across the included studies. Some interventions directly targeted phonological awareness through explicit phonological training activities, whereas others employed broader oral language interventions (e.g., morphosyntactic or vocabulary interventions) with phonological processing measured as a secondary outcome. Improvements in phonological awareness following direct phonological training may reflect specific training effects, whereas improvements following broader language interventions may represent generalised transfer effects. The relatively small number of included studies precluded a reliable moderator analysis comparing these intervention types. Future research should more clearly distinguish between direct and indirect intervention approaches to allow for a more fine‐grained understanding of the mechanisms underlying the observed effects on phonological processing.

Analysis of the intervention approaches reveals that while the taxonomy presented in the introduction (Cabbage and DeVeney 2020; Rvachew and Brosseau‐Lapré 2016) distinguishes between perceptually based, phonologically based, and motorically based approaches, the most used and effective interventions in this review employed integrated approaches. Among the more specific theoretical approaches, input‐oriented auditory perceptual interventions also demonstrated an impact, supporting the theoretical notion that strengthening acoustic‐phonetic representations benefits phonological processing. Phonologically based cognitive‐linguistic approaches, although less frequently used in studies, showed promise for phonological short‐term memory. Motorically based output‐oriented interventions were not present in the data. The review also identified characteristics that appear important, although they could not be analysed. For example, the majority of effective interventions employed direct delivery. Results regarding the use of software suggest that technology is not essential for the effectiveness of interventions. Most interventions did not specify their ingredients or mechanisms of change. This lack of transparency highlights the need for more precise reporting of intervention procedures in future research.

The effects of oral language interventions on output phonology were systematically reviewed and meta‐analysed in an earlier publication of this same group (Kunnari 2024). The findings generally demonstrated medium to large effects on speech production accuracy. However, no significant moderator variables were identified. Additionally, due to an insufficient number of studies, a meta‐analysis could not be performed for other output phonology measures, such as speech intelligibility and phonemic inventory. The relationship between output and input phonology, encompassing speech accuracy and phonological awareness, has been extensively investigated and discussed. Words in the lexicon connect semantic as well as phonetic representations and phonological knowledge to motor plans, and thus, for example, deficits in speech production and phonological processing correlate (Rvachew and Brosseau‐Lapré 2016). Developing speech perception and knowledge of articulatory gestures also supports phonological development and awareness (Fowler 1991; Ivry and Justus 2001; Zhang and McBride‐Chang 2010). Rvachew and Brosseau‐Lapré (2016) describes developmental relationships in developmental speech and/or language disorders and suggests a causal link from speech perception and phonetic representations to both speech accuracy and phonological awareness. Earlier research suggests that speech production unidirectionally predicts phonological awareness (Webster and Plante 1992; Webster and Plante 1995). However, later findings suggest speech production to be only indirectly linked to phonological awareness (Rvachew and Grawburg 2006). Research on the possible causal path from awareness to output is limited (see, however, (Gillon 2000; McNeill et al. 2009; Rvachew et al. 2004)). The findings on phonological awareness and its associations with output phonology are expected to generalise to other areas of phonological processing as well, that is, processing of phonological or sound structure of language, sound‐based representations, and phonological properties of stimuli, when requiring awareness, rapid access, or coding and storage. Beyond spoken language, phonological processing itself predicts reading acquisition and its difficulties (Landerl et al. 2013; Moll et al. 2014).

### 4.4 Strengths and Limitations of the Study

This systematic review and meta‐analysis demonstrate several strengths. First, it provides a detailed characterisation of group design studies and uses multiple moderator variables to analyse the outcomes. Second, given the broad age range of participants, from four to over ten years, the importance of oral language interventions across various language and cultural contexts for improving phonological processing skills is emphasised.

The review also has several limitations that should be acknowledged. First, although grey literature sources (including dissertations and conference proceedings) can provide valuable insights for systematic reviews, our analysis was restricted to peer‐reviewed published literature. This decision was based on two primary considerations: (1) the inclusion of grey literature may introduce publication bias into the analytical framework (Adams et al. 2017); and (2) restricting our scope to peer‐reviewed publications ensured the incorporation of studies meeting established quality standards through the peer review process. This approach strengthened our capacity to address the research questions with methodological rigour and analytical robustness.

Second, we employed common search terms across all COST Action reviews with different foci (i.e., COST Action IS1406 focusing on interventions for children with difficulties learning their first language), which may have limited our capture of specialised phonological processing literature. Third, only articles written in English could be included in the review process, potentially excluding relevant studies published in languages other than English. Finally, four out of 22 studies had sample sizes of fewer than 25 participants, and the small number of studies examining phonological retrieval efficiency resulted in a lack of variability in the data. These limitations may constrain the generalisability of our results.

Additionally, the most recent database search was completed in August 2022, meaning that studies published after this date are not included. This is an inherent consequence of the extended timeline of multi‐site collaborative projects such as the COST Action, where the shared search protocol was established and updated searches were conducted during the Action's funding period. A further update was not feasible within the scope of the present project, and readers should consider this when interpreting the comprehensiveness of the evidence base.

Finally, the included studies covered a broad age range of participants, from four to over ten years. Phonological processing skills undergo substantial developmental changes during this period, and age‐related differences in intervention responsiveness are plausible. However, the small number of included studies precluded again a reliable moderator analysis of age effects, which should be addressed in future research.

### 4.5 Clinical Implications

The findings of this systematic review and meta‐analysis provide direct guidance for clinicians working with children and adolescents with developmental speech and/or language disorders. The demonstrated effectiveness of oral language interventions on phonological processing skills, particularly phonological awareness and phonological short‐term memory, suggests that early intervention can improve these skills, which are relevant for both language and reading development. Clinicians should consider comprehensive programs rather than single‐component interventions, with consistent small‐group delivery emerging as particularly effective. Interventions can also be effectively implemented by non‐specialists, which has implications for service delivery in educational settings, extending the reach of evidence‐based practice. Speech‐language pathologists may therefore consider collaborative models that involve co‐practice and, if necessary, coaching with teachers and/or classroom assistants. The absence of moderators for phonological short‐term memory suggests that clinicians have greater flexibility in implementing interventions across different contexts and delivery methods. The limited evidence for phonological retrieval efficiency interventions highlights a gap in evidence for current practice. Regarding intervention dosage, the studies in this review varied considerably in session duration, frequency, and total intervention period. Most effective interventions involved regular, frequent sessions over several weeks to months. For a more comprehensive analysis of the relationship between intervention dosage and outcomes, readers are directed to the COST Action review focusing specifically on this topic (Frizelle et al. 2021a).

## 5 Conclusions

This systematic review and meta‐analysis synthesise evidence from 22 studies and demonstrates positive effects of oral language interventions on phonological processing skills in children and adolescents with developmental speech and/or language disorders. The results suggest significant and large effect sizes for both phonological awareness and phonological short‐term memory. These findings provide empirical support for the effectiveness of oral language interventions in enhancing phonological processing abilities, offering valuable guidance for clinical practice and future research.

## Conflicts of Interest

The authors declare that they have no conflicts of interest.

## Supporting information




**Supporting File 1**: jlcd70252‐supp‐0001‐SuppMat.docx

**Supporting File 2**: jlcd70252‐supp‐0002‐SuppMat.xlsx

**Supporting File 3**: jlcd70252‐supp‐0003‐SuppMat.docx

**Supporting File 4**: jlcd70252‐supp‐0004‐SuppMat.docx

## Data Availability

Data are available as Supplementary Material.
