# Supplementary material for: Intervention Effects on Phonological Processing in Children With Developmental Speech and/or Language Disorder: A Systematic Review and Meta‐Analysis of Studies With Group Design
Source: Int J Lang Commun Disord. 2026 May 11;61:e70252. doi: 10.1111/1460-6984.70252 (PMC13158719; doi:10.1111/1460-6984.70252)
Supplement: Supplementary file 1 — Supporting File 1: jlcd70252‐supp‐0001‐SuppMat.docx [file JLCD-61-0-s003.docx]

Supplementary Material 1.

Search Strategy for Systematic Reviews of Interventions With Differing Foci Within the COST IS1406 Network

**PubMeD**

MH AND Teaching OR training OR treatment OR “clinical trial” OR intervention OR therapy OR rehabilitation OR remediation OR “special education” OR “dynamic assessment” OR “response to intervention”) AND (“language impairment” OR “language delay” OR “language disorder” OR “language disability” OR “language development disorders” OR dysphasi* OR aphasi* OR “developmental communication disorder”) AND (child* OR preschool* OR adolescen* OR teenage* OR youth) NOT (adult OR deaf OR autis* OR “hearing impairment” OR “Down syndrome” OR “intellectual disability” OR “traumatic brain injury” OR “acquired brain injury” OR “physical disability” OR “learning disability” OR “severe learning difficulties” OR “severe learning difficulty” OR disease) AND ((“2006/01/01”[PDat]: “2015/12/31”[PDat])).

**Web of Science**

TOPIC: ((teaching OR training OR treatment OR “clinical trial” OR intervention OR therapy OR rehabilitation OR remediation OR “special education” OR “dynamic assessment” OR “response to intervention”) AND (language AND (impairment OR delay OR disorder OR disability) OR “language development disorder” OR dysphasi* OR aphasi* OR “developmental communication disorder”) AND (child* OR preschool* OR adolescen* OR teenage* or youth) NOT (adult OR deaf OR autis* OR “hearing impairment” OR “Down syndrome” OR “intellectual disability” OR “traumatic brain injury” OR “acquired brain injury” OR “physical disability” OR “learning disability” OR “severe learning difficulties” OR disease)) 2006–2015 excluding Chemical abstracts.

**ERIC**

((teaching OR training OR treatment OR “clinical trial” OR intervention OR therapy OR rehabilitation OR remediation OR “special education” OR “dynamic assessment” OR “response to intervention”) AND (“language impairment” OR “language delay” OR

“language disorder” OR “language disability” OR “language development disorder” OR dysphasi* OR aphasi* OR “developmental communication disorder”) AND (child* OR preschool* OR adolescen* OR teenage* or youth) NOT (adult OR deaf OR autis* OR

“hearing impairment” OR “Down syndrome” OR “intellectual disability” OR “traumatic brain injury” OR “acquired brain injury” OR “physical disability” OR “learning disability” OR “severe learning difficulties” OR disease))

Setting the year limit as (2006–2015).

**PsychInfo**

((teaching OR training OR treatment OR “clinical trial” OR intervention OR therapy OR rehabilitation OR remediation OR “special education” OR “dynamic assessment” OR “response to intervention”) AND (language AND (impairment OR delay OR disorder OR disability) OR “language development disorder” OR dysphasi* OR aphasi* OR “developmental communication disorder”) AND (child* OR preschool* OR adolescen* OR teenage* or youth) NOT (adult OR deaf OR autis* OR “hearing impairment” OR “Down syndrome” OR “intellectual disability” OR “traumatic brain injury” OR “acquired brain injury” OR “physical disability” OR “learning disability” OR “severe learning difficulties” OR disease)).

**SCOPUS**

(TITLE-ABS-KEY (teaching OR training OR treatment OR “clinical trial” OR intervention OR therapy OR rehabilitation OR remediation OR “special education” OR “dynamic assessment” OR “response to intervention”) AND TITLE-ABS-KEY (“language

impairment” OR “language delay” OR “language disorder” OR “language disability” OR “language development disorder” OR dysphasi* OR aphasi* OR “developmental communication disorder”) AND TITLE-ABS-KEY (child* OR preschool* OR adolescen*

OR teenage* OR youth) AND NOT TITLE-ABS-KEY (adult OR deaf OR autis* OR “hearing impairment” OR “Down syndrome” OR “intellectual disability” OR “traumatic brain injury” OR “acquired brain injury” OR “physical disability” OR “learning disability”

OR “severe learning difficulties” OR disease)) AND SUBJAREA (mult OR medi OR nurs OR vete OR dent OR heal OR mult OR arts OR busi OR deci OR econ OR psyc OR soci) AND PUBYEAR >2005 AND PUBYEAR <2016 AND (EXCLUDE (DOCTYPE,

“le”))
