# Supplementary material for: Intervention Effects on Phonological Processing in Children With Developmental Speech and/or Language Disorder: A Systematic Review and Meta‐Analysis of Studies With Group Design
Source: Int J Lang Commun Disord. 2026 May 11;61:e70252. doi: 10.1111/1460-6984.70252 (PMC13158719; doi:10.1111/1460-6984.70252)
Supplement: Supplementary file 3 — Supporting File 3: jlcd70252‐supp‐0003‐SuppMat.docx [file JLCD-61-0-s002.docx]

Supplementary Material 3. The statistical data used to obtain the individual and average effect sizes

***Experimental and control groups, pre- and post-test: Percentage of Consonants Correct***

| Study | T pre-mean | T pre-SD | T post-mean | T post-SD | T  N | C pre-mean | C pre-SD | C post-mean | C post-SD | C  N | Pre-post corr. | Effect direct. | Standardized  by |
| --- | --- | --- | --- | --- | --- | --- | --- | --- | --- | --- | --- | --- | --- |
| Allen 2013 a | 52.40 | 18.10 | 65.00 | 1.60 | 19 | 54.80 | 18.10 | 58.60 | 1.70 | 16 | 0.70 | P | Post-score SD |
| Allen 2013 b | 54.80 | 18.30 | 60.10 | 1.60 | 19 | 54.80 | 18.10 | 58.60 | 1.70 | 16 | 0.70 | P | Post-score SD |
| Dodd et al. 2008 | 59.00 | 12.94 | 75.10 | 15.00 | 9 | 57.11 | 14.08 | 73.67 | 14.90 | 9 | 0.70 | P | Post-score SD |
| Farquharson et al. 2022 |  |  |  |  |  |  |  |  |  |  |  |  |  |
| Jesus et al. 2019 | 63.68 | 9.67 | 79.85 | 10.99 | 11 | 59.59 | 11.35 | 78.12 | 9.28 | 11 | 0.70 | P | Post-score SD |
| Lousada et al. 2013 | 49.04 | 22.89 | 67.23 | 20.83 | 7 | 42.93 | 18.35 | 50.42 | 19.02 | 7 | 0.70 | P | Post-score SD |
| Murphy 2015 | 77.40 | 12.10 | 77.90 | 11.40 | 10 | 84.10 | 11.90 | 84.30 | 12.60 | 8 | 0.70 | P | Post-score SD |
| Siemons-Lühring et al. 2021 | 81.00 | 11.70 | 89.00 | 8.90 | 16 | 84.30 | 9.30 | 87.5 | 8.10 | 16 | 0.81 | P | Post-score SD |
| Wren et al. 2008 a | 47.50 | 17.40 | 59.50 | 14.60 | 11 | 51.20 | 15.50 | 59.90 | 16.10 | 11 | 0.70 | P | Post-score SD |
| Wren et al. 2008 b | 51.90 | 12.30 | 62.60 | 13.50 | 11 | 51.20 | 15.50 | 59.90 | 16.10 | 11 | 0.70 | P | Post-score SD |

*Note.* T = treated; N = number of participants; C = control; corr. = correlation; direct. = direction; P = positive
