# Supplementary material for: Intervention Effects on Phonological Processing in Children With Developmental Speech and/or Language Disorder: A Systematic Review and Meta‐Analysis of Studies With Group Design
Source: Int J Lang Commun Disord. 2026 May 11;61:e70252. doi: 10.1111/1460-6984.70252 (PMC13158719; doi:10.1111/1460-6984.70252)
Supplement: Supplementary file 4 — Supporting File 4: jlcd70252‐supp‐0004‐SuppMat.docx [file JLCD-61-0-s004.docx]

Supplementary Material 4. Detailed information about the results obtained

**Experimental and control groups, pre- and post-test: Percentage of Consonants Correct**

Table 1

Effect size, heterogeneity and tau-squared

| Model | | Effect size and 95% confidence interval | | | | | Test of null  (2-Tail) | | Heterogeneity | | | | Tau squared | | | |
| --- | --- | --- | --- | --- | --- | --- | --- | --- | --- | --- | --- | --- | --- | --- | --- | --- |
|  | N | Point est. | Std error | S^2^ | Lower limit | Upper limit | Z-value | P-value | Q- value | df (Q) | P- value | I-squared | Tau  squared | Std error | S^2^ | Tau |
| Fixed | 9 | 0.584 | 0.147 | 0.022 | 0.295 | 0.872 | 3.967 | 0.000 | 49.472 | 8 | 0.000 | 83.829 | 1.025 | 0.635 | 0.403 | 1.012 |
| Random | 9 | 0.784 | 0.372 | 0.138 | 0.055 | 1.513 | 2.108 | 0.035 |  |  |  |  |  |  |  |  |

*Note.* There is heterogeneity between the individual effect sizes; N = number of studies; est. = estimate; S^2^ = variance; std = standard

Table 2

Gender and mean age as potential moderator variables

| Covariate | Coefficient | Standard error | 95% Lower | 95% Upper | Z-value | 2-sided P-value |
| --- | --- | --- | --- | --- | --- | --- |
| Intercept | 2.326 | 4.0961 | -5.7021 | 10.3542 | 0.57 | 0.5701 |
| Percentage males treatment | 0.0042 | 0.0391 | -0.0724 | 0.0808 | 0.11 | 0.9148 |
| Mean age treatment group | -0.0303 | 0.0376 | -0.104 | 0.0433 | -0.81 | 0.4193 |
| Statistics for Model 1  Q = 0.88, df = 2, p = 0.6441  Goodness of fit: Test that unexplained variance is zero  Tau² = 1.4089, Tau = 1.1870, I² = 87.15%, Q = 46.68, df = 6, p = 0.0000  Comparison of Model 1 with the null model  Total between-study variance (intercept only)  Tau² = 1.0249, Tau = 1.0124, I² = 83.83%, Q = 49.47, df = 8, p = 0.0000  Proportion of total between-study variance explained by Model 1  R² analogue = 0.00 (computed value is -0.37) | | | | | | |

*Note*. Gender and mean age are unrelated to effect size

Table 3

Setting of intervention as a potential moderator variable

| Group | | Effect size and 95% confidence interval | | | | | Test of null  (2-Tail) | | Heterogeneity | | | | Tau squared | | | |
| --- | --- | --- | --- | --- | --- | --- | --- | --- | --- | --- | --- | --- | --- | --- | --- | --- |
|  | N | Point est. | Std error | S^2^ | Lower limit | Upper limit | Z-value | P- value | Q-value | df (Q) | P- value | I-squared | Tau  squared | Std error | S^2^ | Tau |
| **Fixed effect** |  |  |  |  |  |  |  |  |  |  |  |  |  |  |  |  |
| Preschool | 3 | 1.224 | 0.256 | 0.066 | 0.772 | 1.725 | 4.778 | 0.000 | 38.606 | 2 | 0.000 | 94.819 | 4.074 | 4.665 | 21.759 | 2.018 |
| Preschool and primary | 1 | 0.564 | 0.361 | 0.130 | -0.143 | 1.271 | 1.565 | 0.118 | 0.000 | 0 | 1.000 | 0.000 | 0.000 | 0.000 | 0.000 | 0.000 |
| School | 2 | 0.175 | 0.302 | 0.091 | -0.418 | 0.767 | 0.578 | 0.563 | 0.018 | 1 | 0.895 | 0.000 | 0.000 | 0.258 | 0.067 | 0.000 |
| Clinic | 2 | 0.246 | 0.358 | 0.128 | -0.455 | 0.947 | 0,688 | 0.491 | 0.502 | 1 | 0.479 | 0.000 | 0.000 | 0.368 | 0.136 | 0.000 |
| Total within |  |  |  |  |  |  |  |  | 39.125 | 4 | 0.000 |  |  |  |  |  |
| Total between |  |  |  |  |  |  |  |  | 8.824 | 3 | 0.032 |  |  |  |  |  |
| Overall | 8 | 0.643 | 0.155 | 0.024 | 0.340 | 0.947 | 4.154 | 0.000 | 47.949 | 7 | 0.000 | 85.401 | 1.143 | 0.746 | 0.557 | 1.069 |
| **Mixed effect** |  |  |  |  |  |  |  |  |  |  |  |  |  |  |  |  |
| Preschool | 3 | 2.077 | 1.203 | 1.448 | -0.282 | 4.436 | 1.726 | 0.084 |  |  |  |  |  |  |  |  |
| Preschool and primary | 1 | 0.564 | 0.361 | 0.130 | -0.143 | 1.271 | 1.565 | 0.118 |  |  |  |  |  |  |  |  |
| School | 2 | 0.175 | 0.302 | 0.091 | -0.418 | 0.767 | 0.578 | 0.563 |  |  |  |  |  |  |  |  |
| Clinic | 2 | 0.246 | 0.358 | 0.128 | -0.455 | 0.947 | 0.688 | 0.491 |  |  |  |  |  |  |  |  |
| Total between |  |  |  |  |  |  |  |  | 2.834 | 3 | 0.418 |  |  |  |  |  |
| Overall | 8 | 0.354 | 0.192 | 0.037 | -0.022 | 0.730 | 1.844 | 0.065 |  |  |  |  |  |  |  |  |

*Note.* There is no statistically significant relationship between setting and effect size; N = number of studies; est. = estimate; std = standard; S^2^ = variance

Table 4

Type of practitioner as a potential moderator variable

| Group | | Effect size and 95% confidence interval | | | | | Test of null  (2-Tail) | | Heterogeneity | | | | Tau squared | | | |
| --- | --- | --- | --- | --- | --- | --- | --- | --- | --- | --- | --- | --- | --- | --- | --- | --- |
|  | N | Point est. | Std error | S^2^ | Lower limit | Upper limit | Z-value | P- value | Q-value | df (Q) | P- value | I-squared | Tau  squared | Std error | S^2^ | Tau |
| Fixed effect |  |  |  |  |  |  |  |  |  |  |  |  |  |  |  |  |
| SLT | 5 | 0.211 | 0.203 | 0.041 | -0.186 | 0.609 | 1.041 | 0.298 | 0.539 | 4 | 0.970 | 0.000 | 0.000 | 0.147 | 0.021 | 0.000 |
| SLT / SLP | 3 | 1.243 | 0.239 | 0.057 | 0.774 | 1.712 | 5.197 | 0.000 | 30.574 | 2 | 0.000 | 94.532 | 3.345 | 3.923 | 15.386 | 1.829 |
| Total within |  |  |  |  |  |  |  |  | 37.113 | 6 | 0.000 |  |  |  |  |  |
| Total between |  |  |  |  |  |  |  |  | 10.826 | 1 | 0.001 |  |  |  |  |  |
| Overall | 8 | 0.643 | 0.155 | 0.024 | 0.340 | 0.946 | 4.156 | 0.000 | 47.939 | 7 | 0.000 | 85.398 | 1.141 | 0.745 | 0.555 | 1.068 |
| Mixed effect |  |  |  |  |  |  |  |  |  |  |  |  |  |  |  |  |
| SLT | 5 | 0.211 | 0.203 | 0.041 | -0.186 | 0.609 | 1.041 | 0.298 |  |  |  |  |  |  |  |  |
| SLT / SLP | 3 | 2.165 | 1.095 | 1.199 | 0.020 | 4.311 | 1.978 | 0.048 |  |  |  |  |  |  |  |  |
| Total between |  |  |  |  |  |  |  |  | 3.081 | 1 | 0.079 |  |  |  |  |  |
| Overall | 8 | 0.276 | 0.199 | 0.040 | -0.115 | 0.667 | 1.384 | 0.166 |  |  |  |  |  |  |  |  |

*Note.* There is no statistically significant relationship between practitioner and effect size; N = number of studies; est. = estimate; std = standard; S^2^ = variance

Table 5

Model of delivery as a potential moderator variable

| Group | | Effect size and 95% confidence interval | | | | | Test of null  (2-Tail) | | Heterogeneity | | | | Tau squared | | | |
| --- | --- | --- | --- | --- | --- | --- | --- | --- | --- | --- | --- | --- | --- | --- | --- | --- |
|  | N | Point est. | Std error | S^2^ | Lower limit | Upper limit | Z-value | P- value | Q-value | df (Q) | P- value | I-squared | Tau  squared | Std error | S^2^ | Tau |
| Fixed effect |  |  |  |  |  |  |  |  |  |  |  |  |  |  |  |  |
| Direct | 8 | 0.643 | 0.155 | 0.024 | 0.340 | 0.946 | 4.156 | 0.000 | 47.939 | 7 | 0.000 | 85.398 | 1.141 | 0.745 | 0.555 | 1.068 |
| Indirect | 1 | 0.025 | 0.474 | 0.225 | -0.905 | 0.995 | 0.053 | 0.958 | 0.000 | 0 | 1.000 | 0.000 | 0.000 | 0.000 | 0.000 | 0.000 |
| Total within |  |  |  |  |  |  |  |  | 47.939 | 7 | 0.000 |  |  |  |  |  |
| Total between |  |  |  |  |  |  |  |  | 1.533 | 1 | 0.216 |  |  |  |  |  |
| Overall | 9 | 0.584 | 0.147 | 0.022 | 0.295 | 0.872 | 3.967 | 0.000 | 49.472 | 8 | 0.000 | 83.829 | 1.025 | 0.635 | 0.403 | 1.012 |
| Mixed effect |  |  |  |  |  |  |  |  |  |  |  |  |  |  |  |  |
| Direct | 8 | 0.887 | 0.413 | 0.170 | 0.078 | 1.696 | 2.150 | 0.032 |  |  |  |  |  |  |  |  |
| Indirect | 1 | 0.025 | 0.474 | 0.225 | -0.905 | 0.955 | 0.053 | 0.958 |  |  |  |  |  |  |  |  |
| Total between |  |  |  |  |  |  |  |  | 1.880 | 1 | 0.170 |  |  |  |  |  |
| Overall | 9 | 0.516 | 0.311 | 0.097 | -0.094 | 1.126 | 1.657 | 0.098 |  |  |  |  |  |  |  |  |

*Note.* There is no statistically significant relationship between model of delivery and effect size; N = number of studies; est. = estimate; std = standard; S^2^ = variance
